# Applicability of Age-Based Hunting Regulations for African Leopards

**DOI:** 10.1371/journal.pone.0035209

**Published:** 2012-04-06

**Authors:** Guy Andrew Balme, Luke Hunter, Alex Richard Braczkowski

**Affiliations:** 1 Panthera, New York, New York, United States of America; 2 Wildlife Conservation Research Unit, Department of Zoology, University of Oxford, Oxford, United Kingdom; Australian Wildlife Conservancy, Australia

## Abstract

In species in which juvenile survival depends strongly on male tenure, excessive trophy hunting can artificially elevate male turnover and increase infanticide, potentially to unsustainable levels. Simulation models show that the likelihood of safe harvests can be improved by restricting offtakes to males old enough to have reared their first cohort of offspring to independence; in the case of African leopards, males were ≥7 years old. Here, we explore the applicability of an age-based approach for regulating trophy hunting of leopards. We conducted a structured survey comprising photographs of known-age leopards to assess the ability of wildlife practitioners to sex and age leopards. We also evaluated the utility of four phenotypic traits for use by trophy hunters to age male leopards in the field. Our logistic regression models showed that male leopard age affected the likelihood of survey respondents identifying the correct sex; notably, males <2 years were typically misidentified as females, while mature males (≥4 years) were sexed correctly. Mature male leopards were also more likely to be aged correctly, as were portrait photographs. Aging proficiency was also influenced by the profession of respondents, with hunters recording the lowest scores. A discriminant model including dewlap size, the condition of the ears, and the extent of facial scarring accurately discriminated among male leopard age classes. Model classification rates were considerably higher than the respective scores attained by survey respondents, implying that the aging ability of hunters could theoretically improve with appropriate training. Dewlap size was a particularly reliable indicator of males ≥7 years and a review of online trophy galleries suggested its wider utility as an aging criterion. Our study demonstrated that an age-based hunting approach is practically applicable for leopards. However, implementation would require major reform within the regulatory framework and the hunting industry.

## Introduction

Trophy hunting has the potential to generate substantial financial returns, which may foster tolerance towards large carnivores and enhance opportunities for their conservation outside formally protected areas [1], [2]. However, poorly managed trophy hunting can drive population declines [3], [4]. Felids are especially susceptible to overexploitation due to their complex social systems that depend on the stability of long-term relationships [5]. An artificial increase in turnover and immigration rates can increase contact between unfamiliar individuals and promote intraspecific strife [6], [7]. Unnaturally high turnover among adult males may also increase infanticide, potentially to unsustainable levels [8], [9]. Solitary species appear particularly sensitive to infanticide as females cannot rely on cooperative defence against incoming males [9]. Simulation modelling has suggested that trophy hunting can be sustained by restricting offtakes to males old enough to have reared their first cohort of offspring [8]–[10]. Such an approach eliminates the need for numerical quotas typically derived from unreliable population estimates [11]. Here, we explore the practical application of age-based hunting regulations for leopards *Panthera pardus*.

Leopards contribute 8–20% of gross national trophy hunting income in East and southern Africa [12] and yet, despite their declining status [13], there is little scientific input on the allocation of harvest quotas or the implementation of hunting practices. Although advances in survey methodologies enable accurate estimates of leopard numbers, few authorities employ these techniques in setting quotas [14]. Hunting effort is also frequently distributed unevenly across available leopard range [4], [15]. Such clumped offtake can create localised population sinks that have a disproportionate impact on metapopulation viability [7], [16]. Hunter selectivity additionally appears poor, with female and young male leopards regularly included in trophy harvests, even though it is often illegal to do so [15], [17]. It is difficult to gauge the impacts of such actions, but poorly regulated trophy hunting contributed to high mortality and low recruitment in one intensively-monitored leopard population in South Africa [7] and was instrumental to population declines in Tanzania outside the Selous Game Reserve [4]. More generally, leopards have disappeared from at least 37% of their historical African range [13], prompting the IUCN to recently list the species as Near Threatened [18].

Packer et al. [9] demonstrated that harvesting male leopards ≥7 years old had little impact on population persistence, regardless of offtake. Male leopards have usually left their mothers by 2 years old and can start breeding from 3 years, but typically reach their reproductive peak from 4–6 years, by which time they have held tenure sufficiently long for at least one litter to potentially reach independence [19]. Implementing a strict 7-year age minimum for trophy leopards would dramatically reduce the risk of unsafe harvests despite uncertainties in population sizes. It should also ease pressure from inequitable distribution of quotas as local population recruitment will improve. However, for an age-based system to be applied effectively, hunters must be able to age (and sex) leopards reliably in the field. To date, age determination of leopards has been restricted to the examination of tooth eruption and wear [20], which can only be applied after rather than before an animal is hunted.

In this study, we conduct a structured survey comprising photographs of known-age leopards to assess the ability of wildlife practitioners to sex and age leopards correctly. Contemporary hunters routinely use remotely-triggered cameras to judge the trophy quality of leopards [21]; hence, a photographic survey should provide a reasonable reflection of aging proficiency, as well as demonstrate the age classes hardest to distinguish and the conditions that facilitate accurate aging. We also evaluate the utility of four phenotypic traits for use by trophy hunters to age male leopards in field conditions. The results of the photographic survey indicate the *current* aging ability of hunters while our age determination exercise reveals *potential* aging ability. Finally, we review online trophy galleries to determine whether our aging criteria are pervasive across leopard range.

## Methods

### Photographic Survey

**Figure 1:**
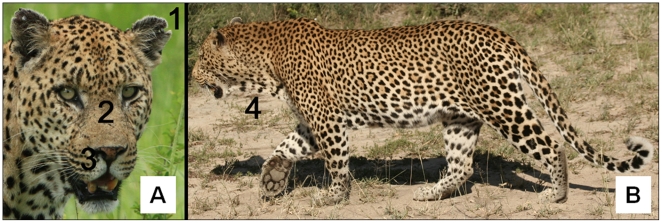
Photograph presentation types used in the survey to test sexing and aging ability. (A) Portrait photo of a 10.3-year male leopard showing the condition of the ears (**1**; score  =  9), facial scarring (**2**; score  =  3) and nose pigmentation (**3**; score  =  pink-spotted); (B) side-profile photo of a 7.5-year male showing dewlap size (**4**; score  =  5) (photo credits: A. Bachelor).

High resolution (minimum 300 ppi) photographs of 31 known-age and sex leopards were sourced from a long-term study in the Sabi Sand Game Reserve (midpoint: 31^o^29' E, 24^o^49' S) adjacent to the Kruger National Park, South Africa. This population has been monitored intensively for >30 years [22], [23] and only photographs of individuals first viewed at <4 months old were included. The survey consisted of two sections; a sexing component comprising 14 photographs of male and female leopards, and an aging component with 44 photographs of male leopards only (Fig. S1). Two types of photograph presentation were used; portrait photos showing the full face of the leopard including the ears (Fig. 1A), and side-profile photos showing the entire body (Fig. 1B). In the first section, participants were asked only to identify photographs as either male or female leopards. In the second section, participants were asked to assign male leopards to one of four age classes: i) <2 years, ii) 2–3 years, iii) 4–6 years, and iv) ≥7 years. The four age classes were represented roughly equally throughout the survey. After thorough pre-testing, the survey was sent to wildlife practitioners from three different professions: i) professional hunters (clients undertaking leopard hunts must be accompanied by a certified professional hunter), ii) photo-tourism guides, and iii) professional felid biologists. Survey participants were randomly selected from the membership lists of national professional hunting (including at least 10 representatives from each of the seven main leopard-hunting countries [15]) and guiding (from South Africa, Zimbabwe and Botswana) associations, and the IUCN Cat Specialist Group (only African-based members) and African Lion Working Group. In addition to sexing and aging leopards, participants were asked to provide information on where they had worked in Africa, the number of years they had worked in their respective fields, and for hunters only, the number of leopards they had successfully hunted. The survey data were analysed anonymously.

We used univariate analyses to explore how the aging proficiency of respondents was affected by profession, age class of leopards, and type of photograph presentation. In addition, we used generalized linear models with a binary logistic response to assess the likelihood of respondents assigning photographs to the correct sex and age categories. Included as predictors were the profession of the respondent (hunter, guide or biologist), respondent experience (number of years), the age class of the photograph (for sexing, five categories were used - the four male age classes plus female), and the type of photograph presentation (portrait, side-profile, and for aging analyses only, paired photos where both portrait and side-profile photos of the same individual at the same age were presented). Respondent identity was included as a random factor. We used odds ratios to measure effect size and the Wald statistic to gauge levels of significance [24]. Model fit was evaluated by assessing residual deviance and likelihood ratio tests [24]. We also applied generalized linear models to hunters separately to determine whether discrepancies existed between this subset of the data and the total sample. We substituted years of experience with number of leopards hunted as a predictor since they were strongly correlated (Pearson’s correlation coefficient  =  0.515, P <0.001).

### Age Determination

We collated 97 paired photographs of 41 known-age male leopards from the Sabi Sand GR displaying facial features and body dimensions. The following phenotypic traits were scored on a sliding scale (Fig. 1): i) extent of facial scarring, from 1 (no scarring evident, the fur above the muzzle appears smooth and glossy) to 5 (heavily scarred, fur has thinned and appears pock-marked); ii) ear condition, from 1 (no wear, ear lobe intact) to 5 (heavily worn, ear lobe extensively notched; the scores for each ear were added, resulting in a maximum score of 10); iii) nose colour, categorised into four classes (pink, pink-grey, pink-spotted, and black; Fig. S2); and iv) dewlap size, scored from 1 (no dewlap visible) to 5 (well-developed dewlap easily recognisable extending from the underside of the maxilla to the upper chest). Two graduate students unfamiliar with the study scored 48 photographs to test repeatability of the method [25]. Their scores were comparable to those given by GB (F_47, 96_  =  37.72, P <0.001, R  =  0.924), suggesting repeatability was high.

To reduce interrelatedness among variables and avoid redundancy in subsequent analyses, we ran a principal component analysis (PCA) based on a correlation matrix of the four phenotypic traits assessed. The factor scores of the first PCA axes that explained >80% of the cumulative data variation were then applied in a discriminant analysis (DA) to determine whether the phenotypic traits could be reliably used to assign male leopards to their respective age classes [26]. The discriminant model was built using a randomly selected 70% of the dataset. The remaining 30% of the data were used to validate the model [26]. Accuracy was assessed by computing the proportion of correctly classified individuals. The likelihood of successful classification can be influenced by the *a priori* probabilities of an observation belonging to a discriminant class; hence, we assumed equal probability of a leopard belonging to any age class (i.e. probability  =  0.25 for each age class [27]). When an individual contributed two or more pairs of photographs from the same age class, we averaged its PCA factor scores before including it in the DA, thus avoiding pseudoreplication [26]. We also conducted a separate DA using dewlap size as the only predictor.

We calculated all analyses and statistical comparisons using SPSS 19.0 (SPSS, Chicago, USA). Significance was measured at P ≤0.05 and two-tailed. We tested all variables for normality and used non-parametric tests where data could not be normalized. We present means with standard error as a measure of precision.

## Results

### Photographic Survey

**Figure 2:**
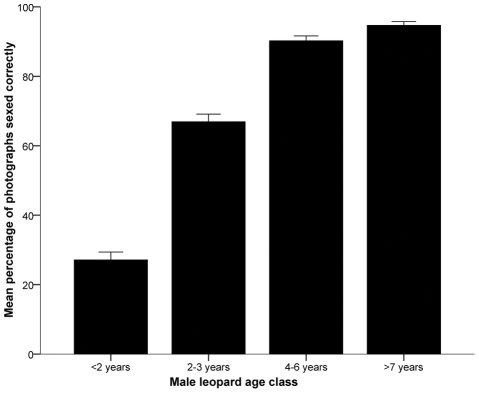
Mean percentage of male leopard photographs in different age classes sexed correctly by survey respondents. Bars show standard error (n  =  225).

The survey was sent out to 357 people and completed by 225 participants (guides: n  =  96, biologists: n  =  59, hunters: n  =  70). Overall, respondents were more successful at sexing leopards (mean percentage correct [MPC]  =  68.95 ± 0.76) than at aging male leopards (MPC  =  47.98 ± 0.57; Z  =  −12.749, P <0.001). Sexing proficiency was similar among professions (guides: MPC  =  70.16 ± 1.08, biologists: MPC  =  68.64 ± 1.27, hunters: MPC  =  67.55 ± 1.62; χ^2^_2_  =  1.712, P  =  0.425) and types of photograph presentation (portrait: MPC  =  69.72 ± 0.94, side profile: MPC  =  67.93 ± 1.09; Z  =  −1.116, P  =  0.264) but varied between age classes (χ^2^_3_  =  404.796, P <0.001). Respondents misidentified 73% of <2 year males as females (Fig. 2). In contrast, >90% of male leopards in the 4–6 year and ≥7 year age classes were sexed correctly (Fig. 2).

Our logistic regression models supported the results of the univariate analyses by suggesting that male leopard age was the only significant factor affecting sexing ability (Table 1). Male leopards in the <2 age class reduced sexing accuracy (odds ratio  =  0.140, χ^2^_1_  =  200.054, P <0.001), while males in the 4–6 year age class (odds ratio  =  4.464, χ^2^_1_  =  77.460, P <0.001) and ≥7 year age class (odds ratio  =  8.592, χ^2^_1_  =  96.932, P <0.001) improved sexing ability. The ratio of residual deviance to degrees of freedom was 0.905, suggesting no over dispersion, and the fitted model differed from the intercept-only model (likelihood ratio χ^2^_11_  =  643.206, P <0.001). Male leopard age remained the only significant predictor when we analysed hunters separately (Table S1).

**Table 1:** Results of generalized linear models assessing the likelihood of survey respondents correctly identifying the sex of leopards in photographs.

Predictor	Wald chi-squared	Degrees of freedom	*P*
Respondent profession	1.161	2	0.560
Respondent experience	3.135	4	0.536
Leopard age class	452.716	4	<0.001
Photograph presentation	2.043	1	0.153

**Figure 3:**
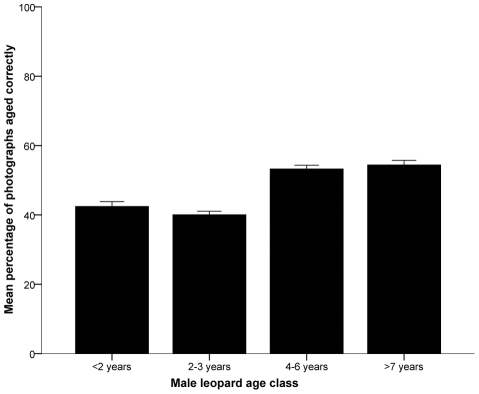
Mean percentage of male leopard photographs assigned to their correct age classes by survey respondents. Bars show standard error (n  =  225).

Respondent aging ability varied among professions (F_2, 224_  =  3.674, P  =  0.027). Hunters (MPC  =  46.04 ± 0.85) performed poorly compared to guides (MPC  =  49.51 ± 1.00; P  =  0.028) but were similar to biologists (MPC  =  47.78 ± 0.93; P  =  0.433). Aging proficiency also varied depending on age classes (χ^2^_3_  =  77.695, P <0.001). Respondents were more successful at distinguishing male leopards in the 4–6 year (MPC  =  53.24 ± 1.06) and ≥7 year (MPC  =  54.40 ± 1.34) age classes than in the <2 year (MPC  =  42.41 ± 1.41) and 2–3 year (MPC  =  40.03 ± 1.04) age classes (Fig. 3). Respondents were also more likely to correctly age portrait photographs (MPC  =  60.18 ± 0.93) than side-profile (MPC  =  42.80 ± 0.76) or paired photographs (MPC  =  35.68 ± 1.18; χ^2^_2_  =  185.297, P <0.001).

Our logistic regression models suggested that respondent profession, male leopard age, and photograph presentation were all significant predictors of aging ability (Table 2). Hunters were the worst affected of the three professions, significantly reducing the likelihood of a correct answer (odds ratio  =  0.858, χ^2^_1_  =  5.747, P  =  0.017). Male leopards in the 4–6 age class (odds ratio  =  1.602, χ^2^_1_  =  52.125, P <0.001) and the ≥7 year age class (odds ratio  =  1.681, χ^2^_1_  =  63.301, P <0.001) were more likely to be aged correctly, as were portrait photographs (odds ratio  =  1.988, χ^2^_1_  =  188.517, P <0.001). Model fit was good (ratio of residual deviance to degrees of freedom  =  1.492; likelihood ratio χ^2^_11_  =  466.792, P <0.001). Male leopard age and photograph presentation remained as significant predictors when we analysed hunters separately (Table S2).

**Table 2:** Results of generalized linear models assessing the likelihood of survey respondents assigning photographs of male leopards to their correct age class.

Predictor	Wald chi-squared	Degrees of freedom	*P*
Respondent profession	6.003	2	0.050
Respondent experience	2.446	4	0.654
Leopard age class	125.634	3	<0.001
Photograph presentation	312.133	2	<0.001

### Age Determination

**Figure 4:**
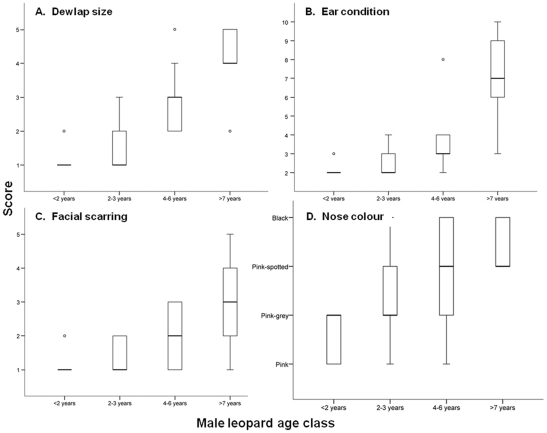
Relationships between four phenotypic traits and age classes of male leopards. Boxes indicate the lower, median and upper quartiles, vertical lines represent the sample minimum and maximum, and open circles correspond to outliers.

All four phenotypic traits varied between age classes (dewlap size: χ^2^_3_  =  53.309, P <0.001; facial scarring: χ^2^_3_  =  29.396, P <0.001; ear condition: χ^2^_3_  =  47.112, P <0.001; nose pigmentation: χ^2^_3_  =  18.018, P <0.001). Post hoc analyses revealed significant differences between the ≥7 year age class and all other age classes for dewlap size (P  =  0.002) and ear condition (P <0.001). Male leopards ≥7 years old generally had well developed dewlaps; only one individual in this age class (n  =  15) had a dewlap score of less than 4 (Fig. 4A). Ear condition varied considerably among ≥7 year old leopards but usually at least one ear showed some degree of wear, whereas in younger age classes there was little wear (Fig. 4B). Facial scarring tended to increase with age but there was considerable overlap between the 4–6 year and ≥7 year age classes (Fig. 4C). Only black and pink-spotted noses were observed in the ≥7 year age class, but these pigmentation categories were also found in other age classes (Fig. 4D).

The first two factors of the PCA explained >80% of the data variation and were included in the DA. The first factor primarily represented dewlap size, ear condition, and facial scarring and accounted for 70% of variance (eigenvalue  =  2.814). The second factor mainly represented nose pigmentation and only accounted for 16% of variance (eigenvalue  =  0.649). Only factor 1 contributed significantly to the DA (Wilk’s Lambda  =  0.137, F_3, 45_  =  94.637, P <0.001), not factor 2 (Wilk’s Lambda  =  0.948, F_3, 45_  =  0.825, P  =  0.487). Similarly, only the discriminant function strongly correlated to factor 1 (structure correlation coefficient  =  1.000) was statistically significant (Wilk’s Lambda  =  0.130, χ^2^_6_  =  91.885, P <0.001), explaining 99% of the discriminatory power of the model (eigenvalue  =  6.309). Overall, our general model classified 67% of cases correctly, with success rates ranging from 50% for the 4–6 year age class to 83% for the ≥7 year age class (Table 3). Our validation model showed a 7% reduction in overall success but classification rates for the ≥7 year age class remained high (100%). Classification rates for our reduced discriminant model using dewlap size as the only predictor were similarly high for male leopards ≥7 year old (91–100%).

**Table 3:** Relative success rates of discriminant models classifying male leopards into their respective age classes based on principal component factor scores for dewlap size, ear condition, and facial scarring.

	Full model (predictors: dewlap size, ear condition, facial scarring)		Reduced model (predictors: dewlap size)	
**Age class**	**General**	**Validation**	**General**	**Validation**
<2 years	66.7 (15)	83.3 (6)	84.6 (13)	100.0 (8)
2–3 years	64.3 (14)	50.0 (6)	25.0 (16)	25.0 (4)
4–6 years	50.0 (8)	20 (5)	27.3 (11)	50.0 (2)
≥7 years	83.3 (12)	100.0 (3)	90.9 (11)	100 (4)
Total	67.3 (49)	60.0 (20)	54.9 (51)	77.8 (18)

Discriminant functions were built using 70% of the data with the remainder used to validate models. Rates are presented as percentages with samples sizes in parentheses.

## Discussion

### Sexing Leopards

The superior ability of respondents to sex rather than age leopards is unsurprising given the genus *Panthera* exhibits the most striking sexual dimorphism among extant wild felids [28]. In our study area, adult male leopards weigh at least 60% more than females [29] and the same is true across most of the species’ range [30]. Such marked size dimorphism extends to the cranial morphology (males have longer and broader skulls than females), body length, neck circumference, chest girth, and shoulder height of leopards (Table S3). Although it is difficult to gauge body size from photographs, the relative dimensions are clearly apparent. Our survey showed that almost all respondents could differentiate mature (≥4 years) male leopards from females. In contrast, there was considerable confusion in distinguishing females from <2 year old males. At this age, male leopards superficially resemble females; they are a similar size (Table S3) and lack many of the distinctive features of adult males (e.g. well-developed chest and neck musculature, a prominent dewlap, etc.). None of the photographs in our survey displayed primary sexual characters (i.e. the scrotum or nipples). Although hunters may be able to use the external genitalia to distinguish young male leopards from females, the scrotum tends to become more conspicuous with age, and it may not be immediately obvious in males <2 years old (G. A. Balme personal observation).

### Aging Male Leopards

Respondents performed poorly at aging male leopards, with less than 50% of photographs classified correctly. Hunters recorded the lowest scores, which presumably reflects the relative amount of time they spend observing leopards. A professional hunter will rarely lead >5 leopard hunts a year (and usually only 1–3 depending on the country), whereas photo-tourism guides may view leopards weekly, or in some areas like our study site, daily [22]. Biologists also typically do not observe leopards on a regular basis, although they are at least likely to be familiar with the aging cues associated with felids since many are shared among species [31]. Importantly, our multivariate analyses showed that aging (and sexing) ability was not related to levels of experience; hence, with appropriate training, it should be possible to educate hunters and other wildlife practitioners to age leopards more reliably [32], [33].

Portrait photographs appeared to increase aging proficiency. This may be due to the larger number of aging cues exhibited in portrait photos compared to side-profile photos. Portrait photographs show the condition of the ears, facial scarring, nose pigmentation, the relative ‘broadness’ of the skull, and occasionally tooth wear of leopards. In contrast, side-profile photos only show relative body dimensions and dewlap size (though our age determination analyses suggest this should be sufficient). We expected that paired photos should perform the best as they present the most cues but this was not the case in our study. There is no obvious explanation for this result except perhaps that respondents focused mainly on the larger, side-profile photograph in paired examples, indicating a potential flaw in the survey design.

Respondents were more successful at distinguishing leopards in adult age classes (4–6 years and ≥7 years) than subadults (2–3 years) or juveniles (<2 years). Our discriminant models confirmed that ≥7 year old males were the easiest to identify but, contrary to the results of the survey, 4–6 year males registered the most misclassifications. The classification rate recorded for this age class in the general model (50%) was nevertheless similar to that achieved by respondents in the survey (53%), suggesting that our aging methodology is unlikely to improve hunters’ ability to recognise 4–6 year old leopards. However, there appears considerable scope for improvement among the other age classes. Classification rates in the full model were significantly higher for <2 year, 2–3 year, and ≥7 year males than the respective scores attained by respondents in the survey.

The condition of the ears, facial scarring, and dewlap size were all related to the significant discriminant function in our model; only nose colour appeared a poor predictor of male leopard age. It is worth noting that we did not measure the extent of nose pigmentation quantitatively as was done for lions *Panthera leo* [8]; we simply categorised the overall colour of noses visually. This method is admittedly susceptible to human error or subjective differences of opinion. However, our goal was to determine whether hunters could use nose colour and the other phenotypic traits assessed as mechanisms to reliably age leopards in the field. Moreover, we demonstrated that repeatability among observers was high. Therefore, we feel that our method of visual assessment was valid within the context of our study.

**Figure 5:**
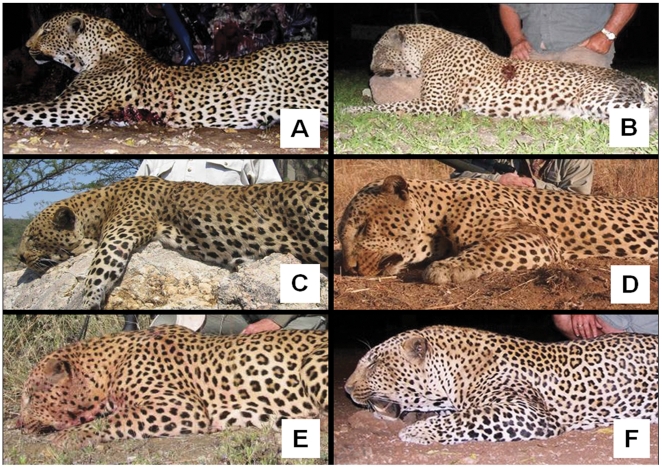
Examples of male leopard trophies exhibiting well-developed dewlaps from the main leopard hunting countries . (A) Botswana; (B) Mozambique; (C) Namibia; (D) Tanzania; (E) Zambia; (F) Zimbabwe. These countries (with the addition of South Africa) are permitted under the Convention for the International Trade in Endangered Species (CITES) to export >100 leopard trophies annually from hunting [15].

Most leopards are hunted over baits at a distance of 50–80 m in low light conditions (regulations vary between countries regarding the legal timing of hunts and use of artificial lighting [34], [35]). It may be impractical for hunters to assess the facial characteristics of leopards under such circumstances (although the increasing use of remotely-triggered cameras by hunters should facilitate this). However, the dewlap is a conspicuous feature easily discernible from a distance. Our analyses showed that there was little overlap in dewlap size between ≥7 year males and younger age classes. Furthermore, our reduced discriminant model demonstrated that dewlap size on its own was a reliable predictor of male leopard age. We identified males with well-developed dewlaps in all of the main leopard hunting countries during our online review of trophy galleries (Fig. 5). The ages of trophy leopards were not known but, in instances where these cues were visible, the condition of the ears and facial scarring often correlated with dewlap size. Our results therefore suggest that at least in the savanna regions of East and southern Africa where most leopards are hunted [15], dewlap size could be used as a practical criterion to identify suitably-aged individuals. Further site-specific research is nevertheless required regarding the relationship between dewlap size and age, particularly from forest and semi-arid environments where leopard morphology varies considerably from savanna habitats [30]. Dewlap size is related to physical condition in some species (e.g. Bali cattle *Bos javanicus* [36]) and the same may be true in leopards (Fig. S3). However, this will not affect its usefulness in improving hunter selectivity; some old leopards in poor condition may be overlooked but it will not result in younger animals being harvested.

### Conservation Implications and Recommendations

It is illegal in most countries (with South Africa being a notable exception) to hunt female leopards, but compliance appears low. Genetic analyses showed that females comprised 27% of 77 leopard trophies shot in Tanzania between 1995 and 1998, even though only males are legally harvested there [17]. Our review of trophy galleries also revealed a remarkable number of hunted female leopards on hunting company websites (and this is an optimistic representation of trophy quality as operators are likely to display their best specimens for marketing; Fig. S4). According to our survey results, stipulating a minimum trophy age of ≥7 years for male leopards will essentially eliminate the possibility of hunters mistakenly harvesting females. Several polygynous felids are resilient to disturbance if the prime reproductive female life-stage remains intact [37], [38]. Since one male can mate with numerous females, fewer males are required to maintain the same levels of reproduction. Hunting adult females carries the additional risk of dependent cubs dying when their mother is killed [6]. Male leopards also disperse over greater distances than females [29], enabling more efficient replacement of hunted individuals. A population viability analysis conducted for the South African leopard population showed that risk of extinction almost doubled when females were included on quota [34]. The ‘7-year age rule’ for leopards was also derived under the assumption of a male-only harvest [9].

The overall predictive power of our discriminant models (55–67%) was mediocre (although they were at least as accurate as others proposed to age carnivores [26], [39], [40]), but confidence levels for discerning males ≥7 years old were high (83–100%). This suggests that a minimum age threshold for leopard trophies could practically be applied to ensure sustainable hunting. However, it would require strict enforcement by government authorities to be effective [41]. The age of every leopard trophy will have to be independently validated. The same criteria used by hunters to estimate leopard age can be used by authorities to evaluate trophies (with the addition of tooth wear; see Fig. S5). Unsuitable trophies (a female leopard or male <7 years old) can be confiscated [4]. Alternatively, hunting operators that take unsuitable trophies could be penalised by a reduction in quota the following year, while operators that harvest suitably-aged individuals can be rewarded with an increase in quota the following season. Such an incentive-based approach has been used to regulate trophy hunting of lions in Niassa National Reserve in northern Mozambique [42]. Hunting offtakes in Niassa have subsequently declined to sustainable levels, trophy quality has improved and the local lion population has increased [42]. The production of a comprehensive leopard aging guide (similar to that compiled for lions [31]) should help improve the aging ability of hunters. Leopard aging techniques could also be incorporated in the curricula of appropriate hunting courses with the successful completion of an examination a prerequisite for licensing (as is the case in the United States for mountain lions *Puma concolor*; http://wildlife.state.co.us/Hunting/HunterEducation/MtnLionEduc/Pages/MountainLionExam.aspx, accessed November 2011).

The implementation of age-based hunting regulations for leopards would not necessarily disadvantage hunters. Provided age-limits are strictly adhered to, the number of animals available to hunt (≥7 year old males comprised roughly 8% of our study population; G. A. Balme unpublished data) exceeds that proposed for sustainable population-based quotas (3.8% of the population [5]). Indeed, Whitman et al. [8] showed that the cumulative number of ‘high-quality’ lion trophies harvested was greatest when recommended age minimums were adhered to. The minimum length of leopard safaris (mean  =  10–14 days [43]) could also be extended to accommodate the increased selectivity demanded of hunters. Individual operators stand to gain as their clients are typically charged a daily rate regardless of whether hunts are successful.

The results of our study have implications that extend beyond hunting. Age determination is an important prerequisite for most large carnivore research. Variation in life history traits is closely related to age, as is the social behaviour and spatial patterns of individuals [6], [29]. Our aging criteria provide an accurate and non-invasive method for aging leopards easily replicable across sites. Camera-trap surveys are widely used to estimate leopard abundance [14], [44], [45] and our methodology enables a robust assessment of population structure as well as size. It can similarly be used to estimate the age of telemetered individuals or leopards captured during problem-animal-control operations. Such knowledge is vital to understanding population dynamics and informing management activities.

For trophy hunting to serve as a conservation tool, it is essential that it be conducted in a manner that is scientifically robust and sustainable in the long term [46]. We demonstrate that the potential exists for the practical application of an age-based hunting system for leopards, which would reduce the risks of over-harvest and deleterious impacts on hunted populations. Hunting operators also stand to benefit as trophy quality is likely to improve (without necessarily an associated reduction in quota) and longer hunts can be offered. However, the successful implementation of an age-based hunting approach requires major reform within the regulatory framework and among the hunting industry. It remains to be seen if such changes are realistic.

## Supporting Information

Figure S1 **Survey used to test the ability of wildlife practitioners to sex and age leopards.** The survey comprises three sections: 1) respondents must sex photographs of male and female leopards, 2) respondents must assign single photographs of male leopards to one of four age classes (<2 years, 2–3 years, 4–6 years, or ≥7 years), and 3) respondents must assign paired photographs of the same individual male leopard to their respective age class.
(PDF)

Figure S2 **Examples of nose colour categories used in the age determination analyses.** (A) 11-month male: nose colour category  =  pink; (B) 2.8-year male: nose colour category  =  pink-grey; (C) 5.3-year male: nose colour category  =  pink-spotted; (D) 9.0-year male: nose colour category  =  black.
(TIF)

Figure S3 **Effect of body condition on dewlap size in male leopards.** The same individual male leopard camera-trapped in July 2009 (A) and August 2010 (B) in Niassa National Reserve, Mozambique (Photo credits: Niassa Carnivore Project). It is unknown what caused the deterioration in condition.
(TIF)

Figure S4 **Examples of leopard trophies from different countries exhibited on hunting company websites that are likely females or <2 year males.** (A) Botswana; (B) Mozambique; (C) Namibia; (D) Tanzania; (E) Zambia; (F) Zimbabwe.
(TIF)

Figure S5 **Protocol for collecting data from trophy hunted lion and leopard.**
(TIF)

Table S1 **Results of generalized linear models assessing the likelihood of professional hunters correctly identifying the sex of leopards in survey photographs.**
(DOC)

Table S2 **Results of generalized linear models assessing the likelihood of professional hunters assigning photographs of male leopards to their correct age class.**
(DOC)

Table S3 **Morphological measurements of radio-collared leopards from the Phinda-Mkhuze Complex (PMC) in northern KwaZulu-Natal, South Africa** [**19**] **.**
(DOC)
