# Supplementary material for: Applicability of Age-Based Hunting Regulations for African Leopards
Source: PLoS One. 2012 Apr 6;7(4):e35209. doi: 10.1371/journal.pone.0035209 (PMC3320874; doi:10.1371/journal.pone.0035209)
Supplement: Figure S1 — Survey used to test the ability of wildlife practitioners to sex and age leopards. The survey comprises three sections: 1) respondents must sex photographs of male and female leopards, 2) respondents must assign single photographs of male leopards to one of four age classes (<2 years, 2–3 years, 4–6 years, or ≥7 years), and 3) respondents must assign paired photographs of the same individual male leopard to their respective age class. (PDF) [file pone.0035209.s001.pdf]

SECTION 1: SEXING LEOPARDS

Numbers 1-14 include photographs of both male and female leopards. Please specify the sex of the leopard. It is not necessary to age individuals. Each photograph represents a unique individual and must be assessed independently.

1)

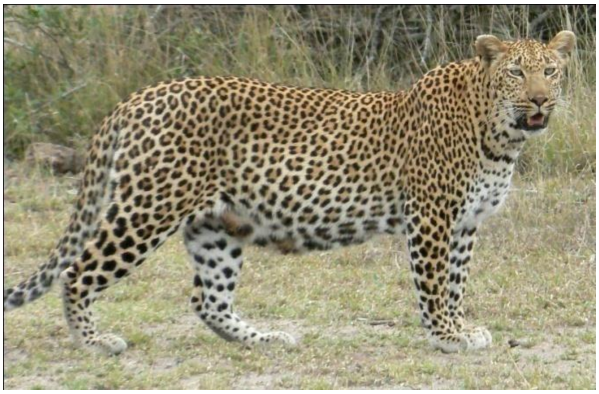

2)

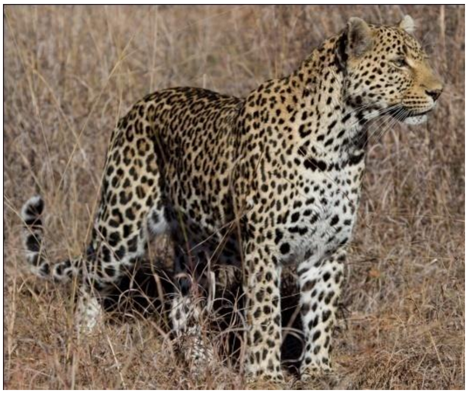

3)

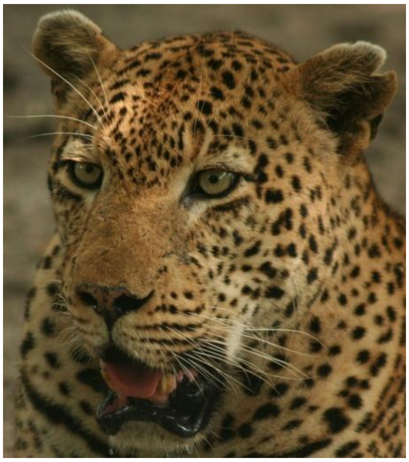

4)

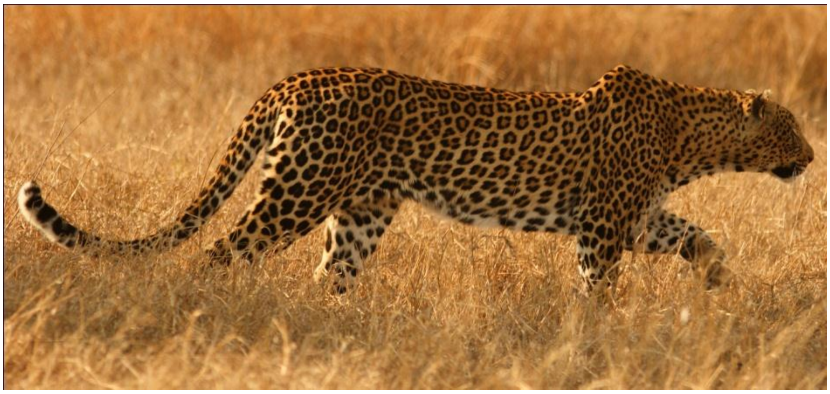

5)

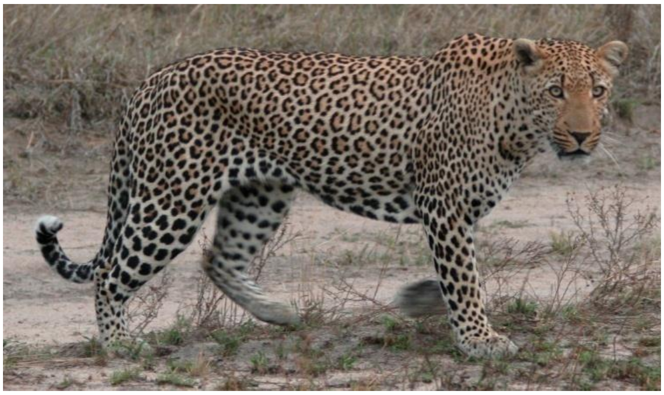

6)

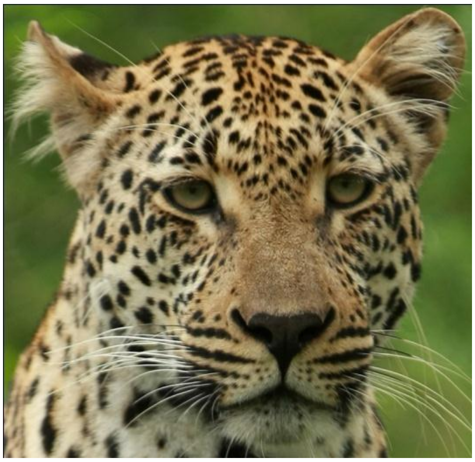

7)

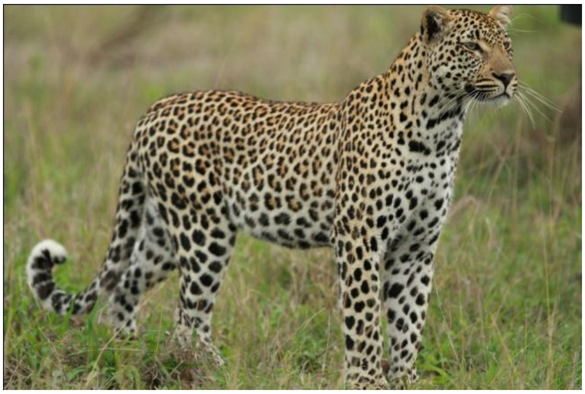

8)

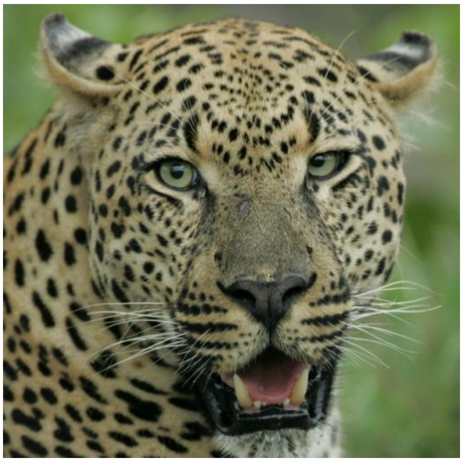

9)

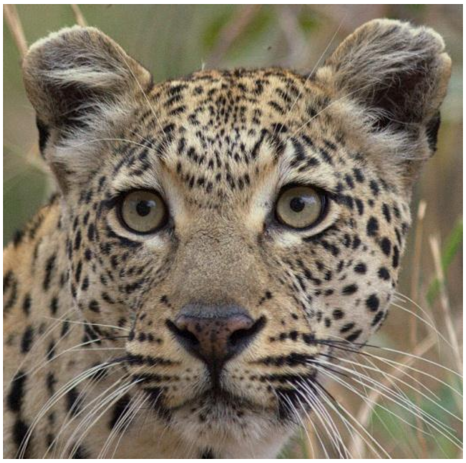

10)

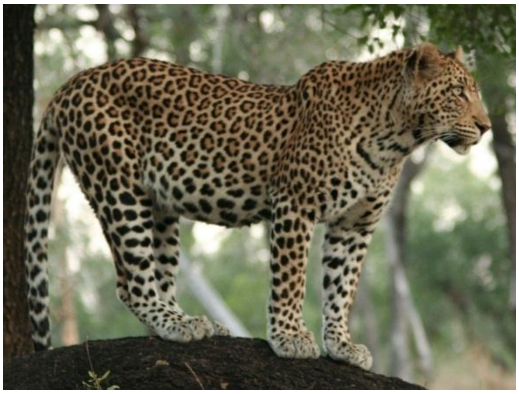

11)

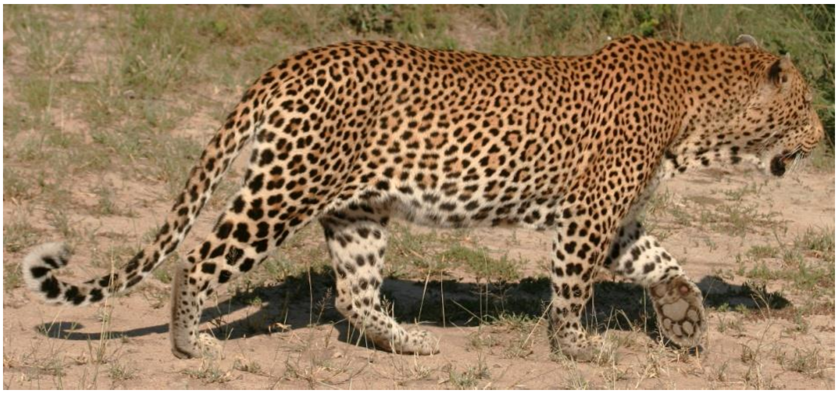

12)

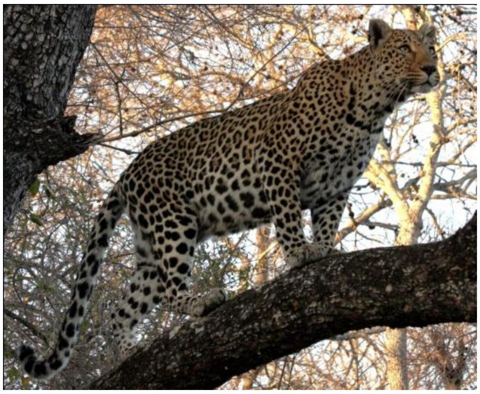

13)

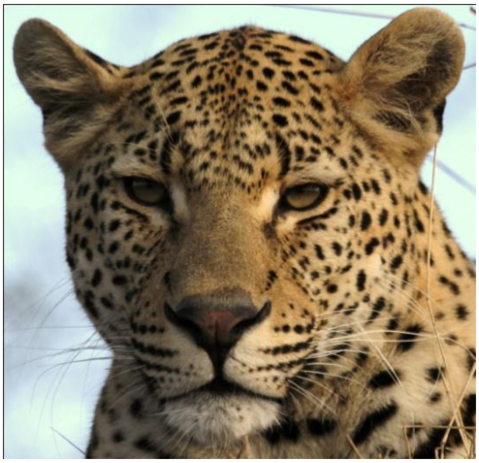

14)

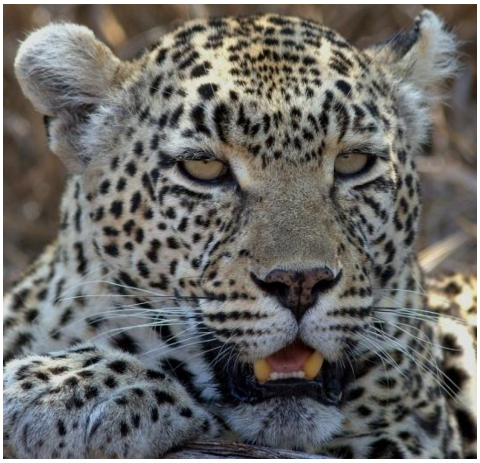

SECTION 2: AGING MALE LEOPARDS

Numbers 15-44 include photographs of male leopards only. Please classify individuals into one of four age categories:
<2 years, 2-3 years, 4-6 years, ≥7 years.
Each photograph represents a unique individual and must be assessed independently.

15)

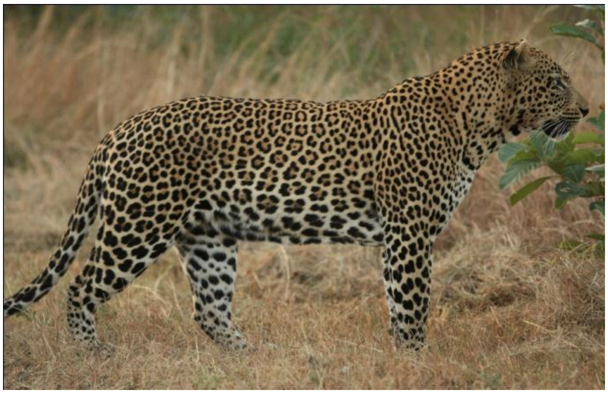

16)

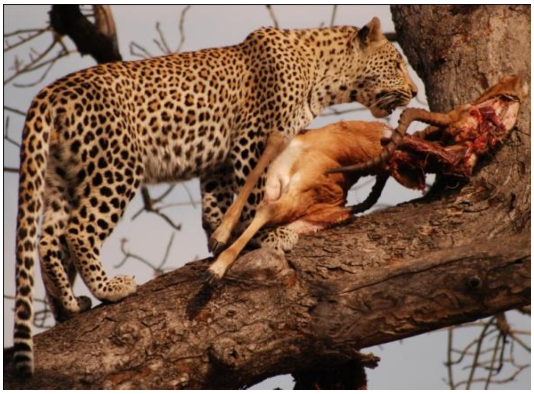

17)

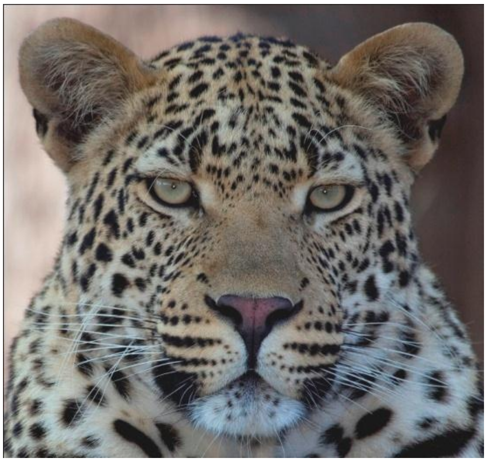

18)

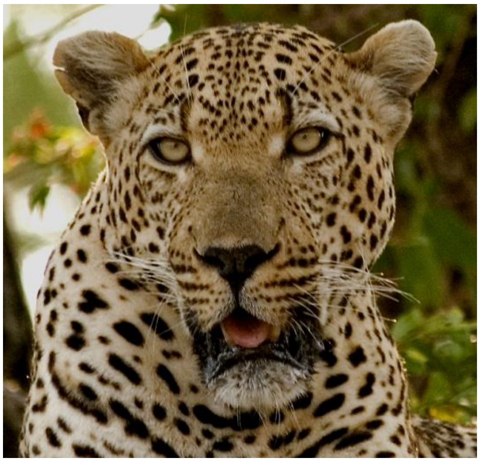

19)

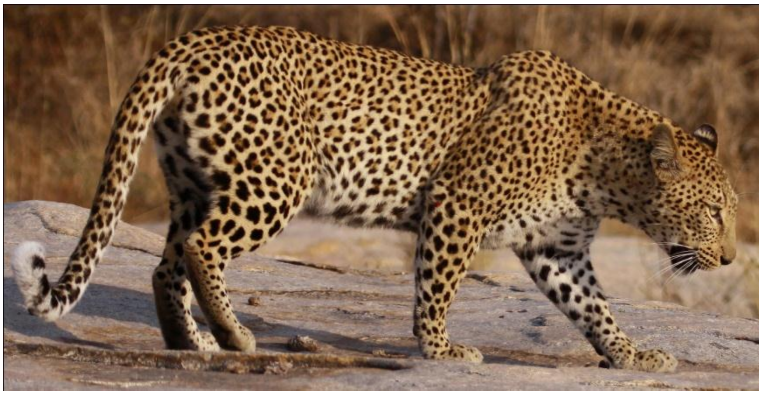

20)

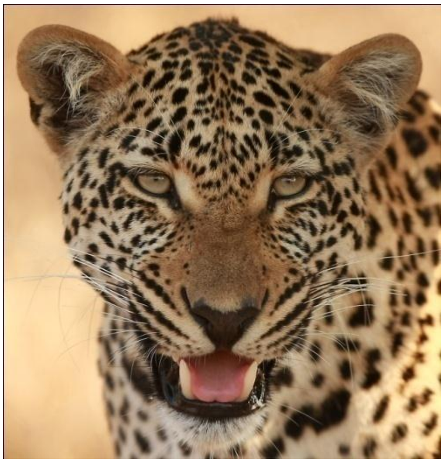

21)

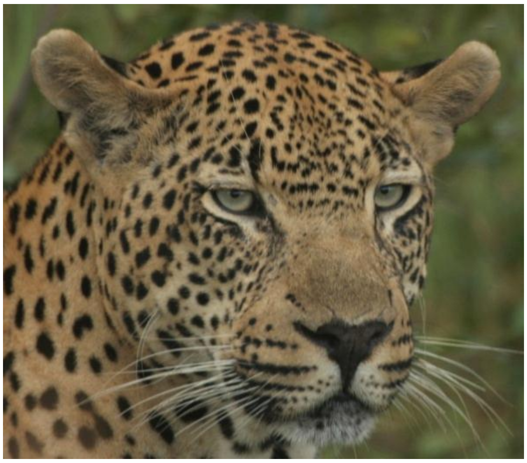

22)

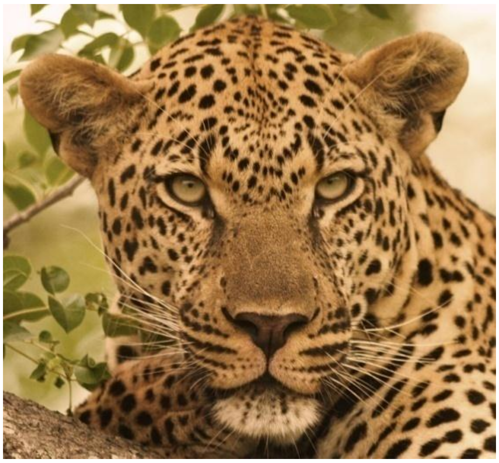

23)

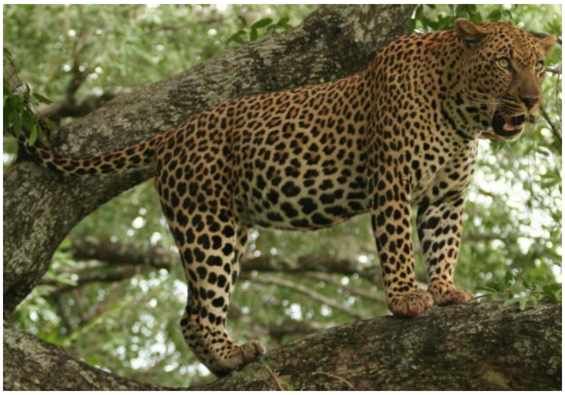

24)

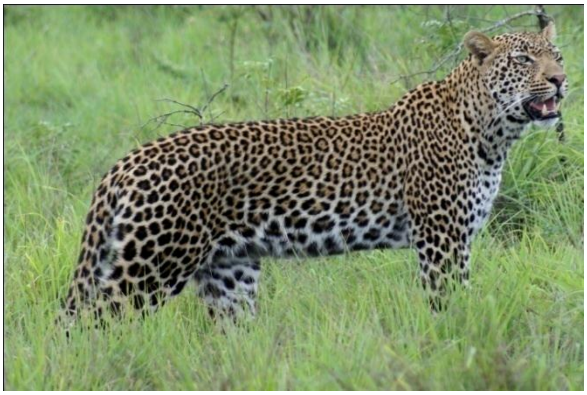

25)

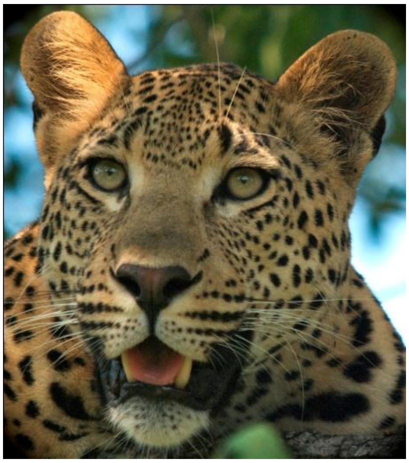

26)

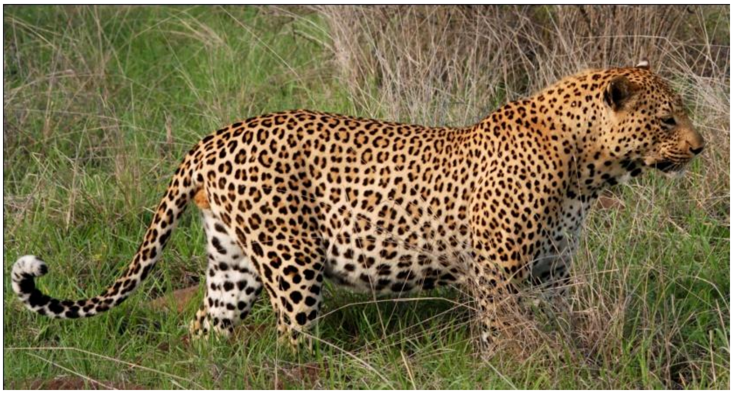

27)

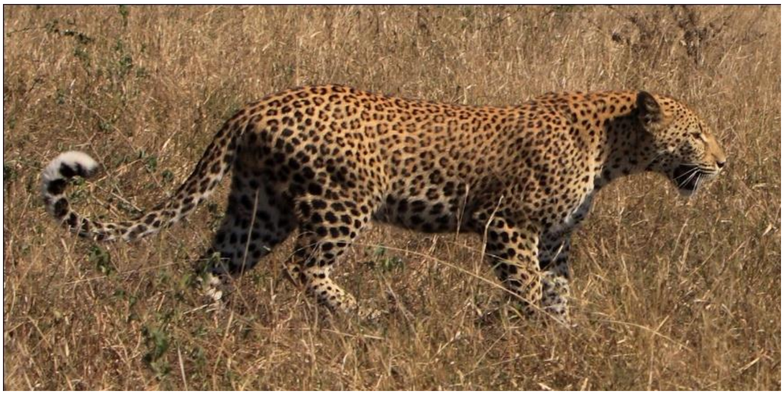

28)

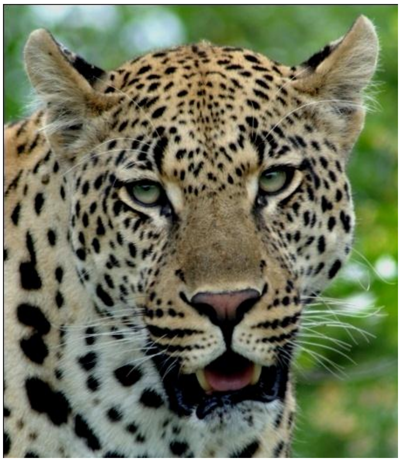

29)

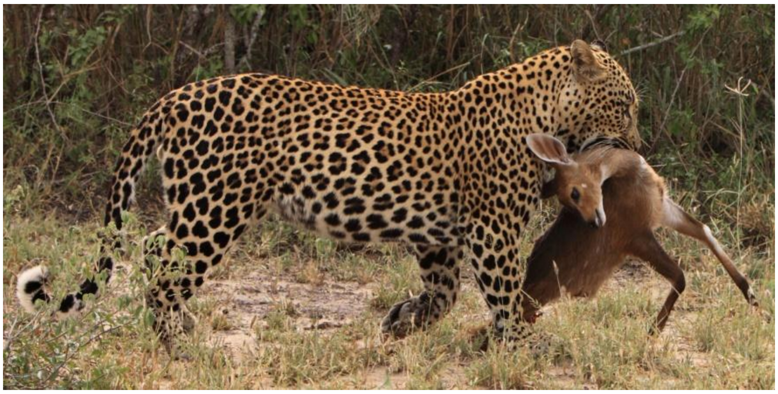

30)

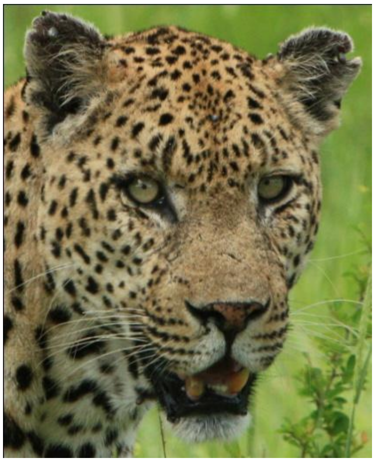

31)

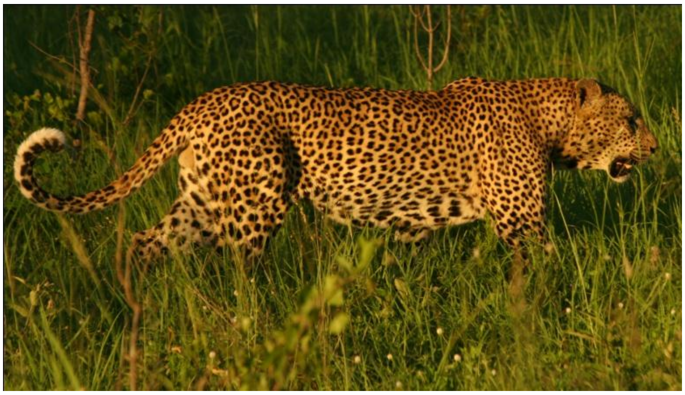

32)

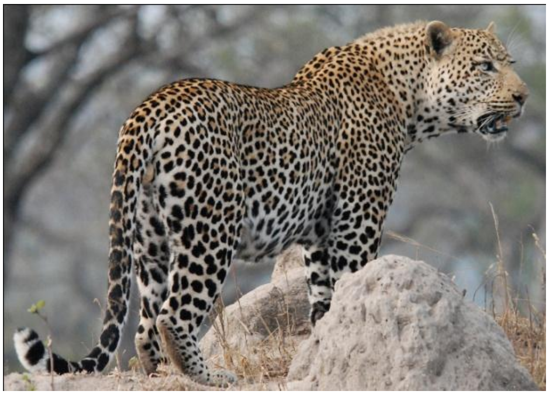

33)

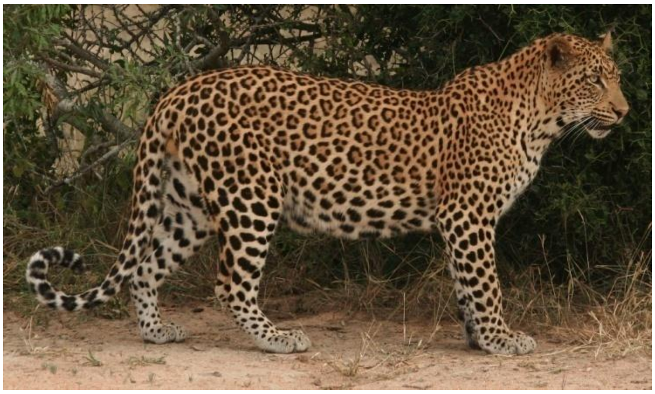

34)

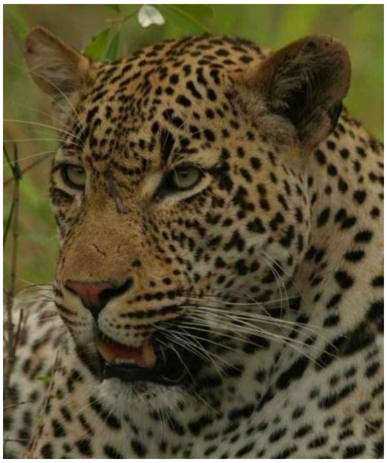

35)

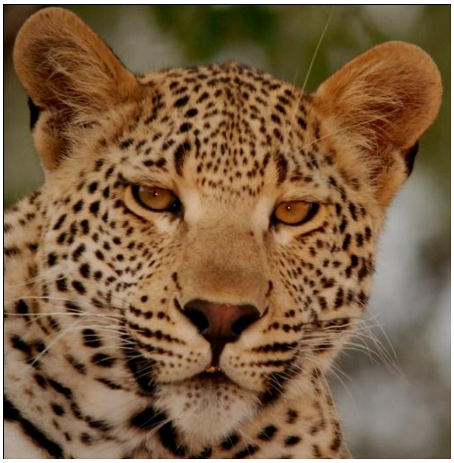

36)

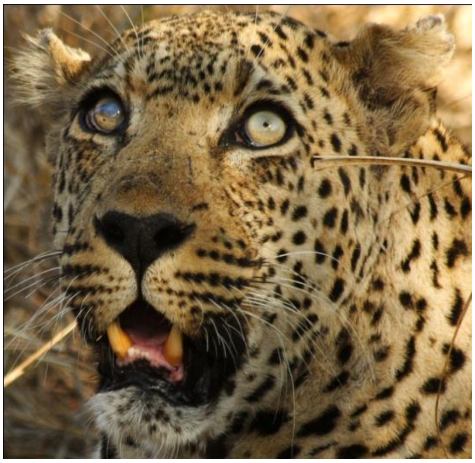

37)

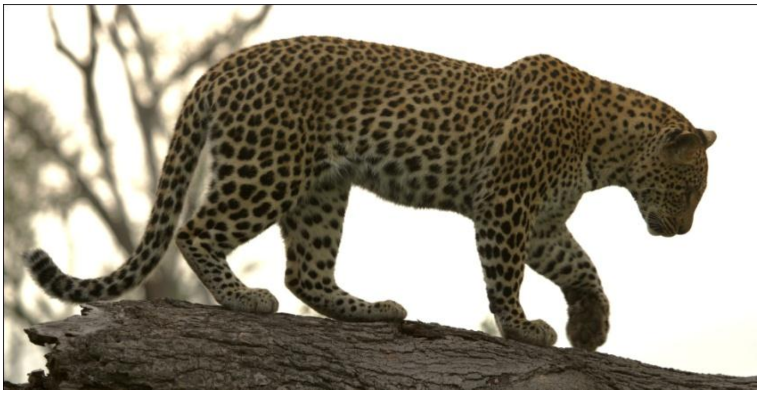

38)

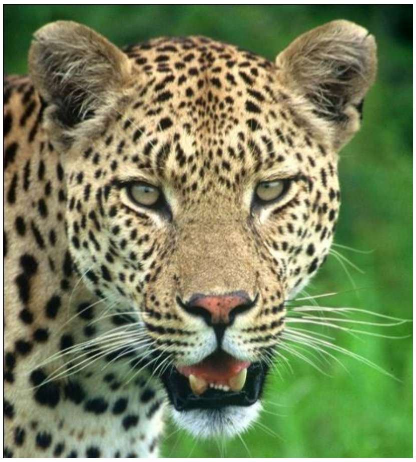

39)

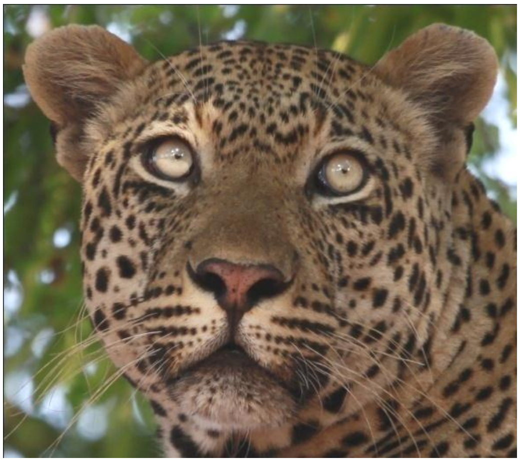

40)

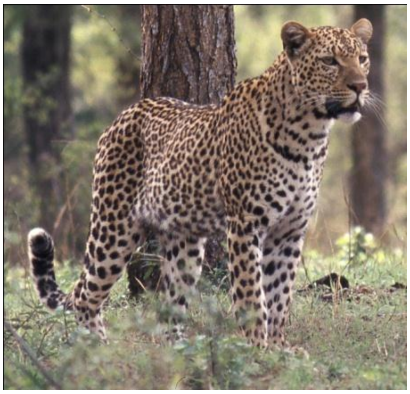

41)

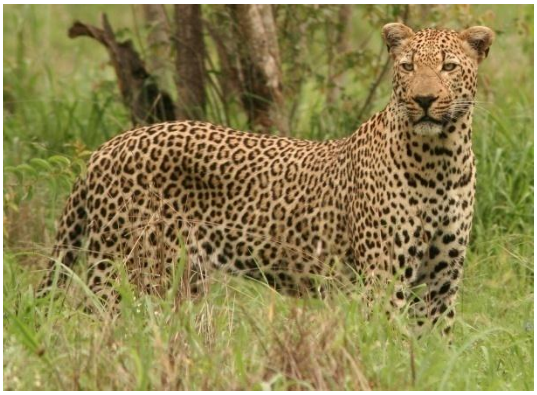

42)

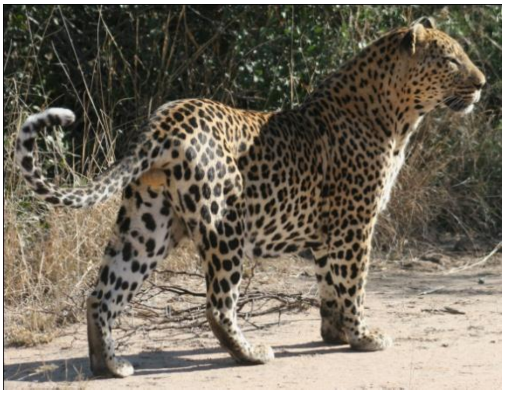

43)

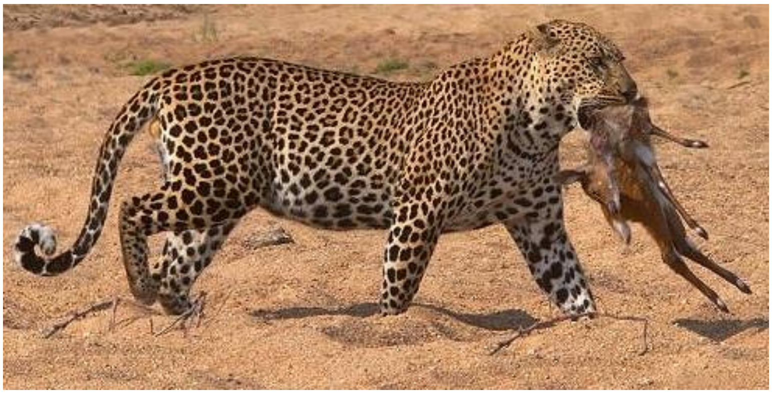

44)

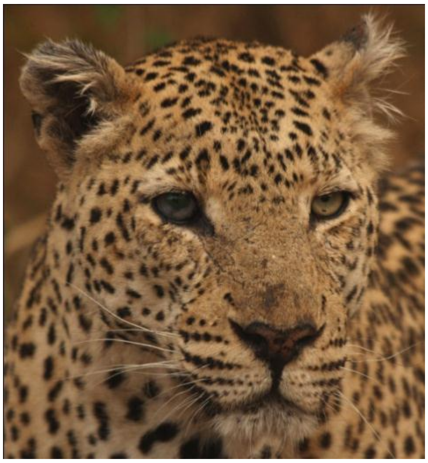

SECTION 3: AGING MALE LEOPARDS USING PAIRED PHOTOGRAPHS

Numbers 45-51 include paired photographs of the same individual male leopard taken at the same age. Please classify individuals into one of four age categories:
<2 years, 2-3 years, 4-6 years, ≥7 years.

45)

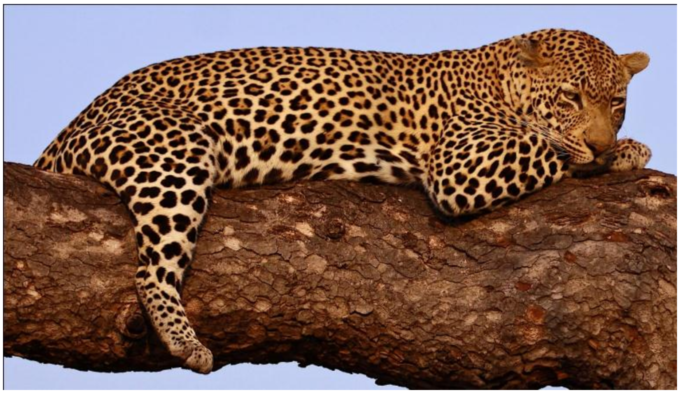

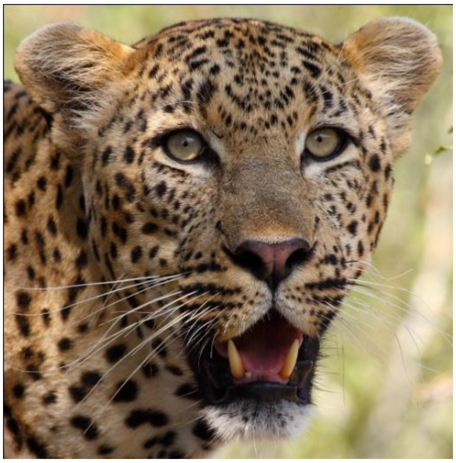

46)

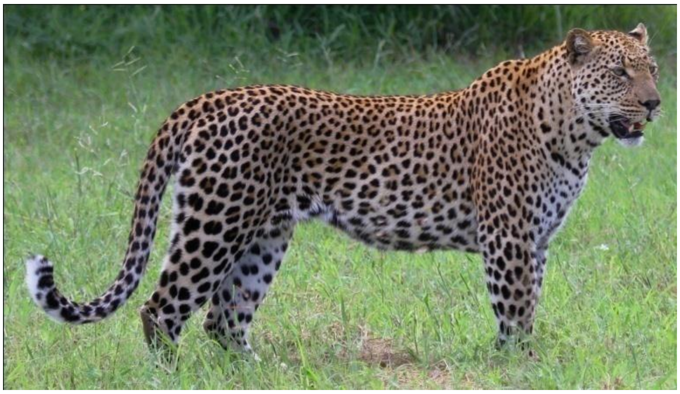

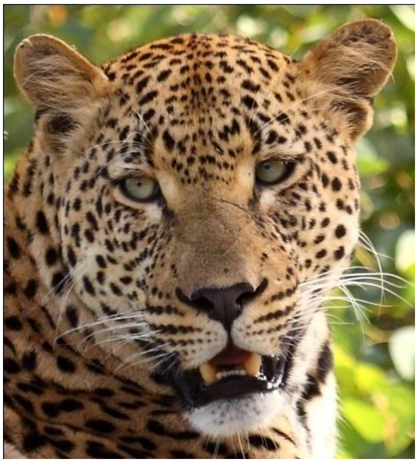

47)

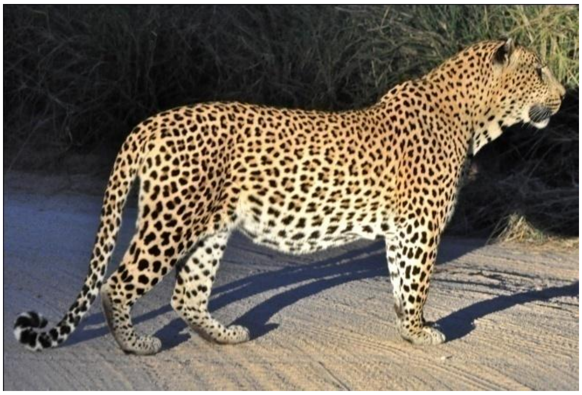

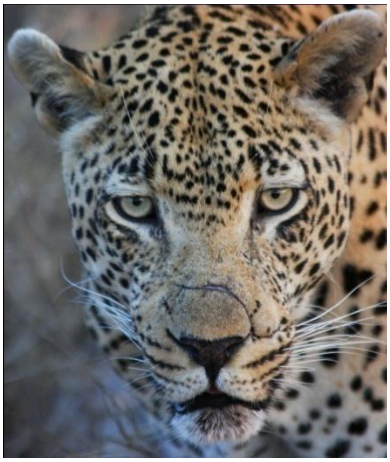

48)

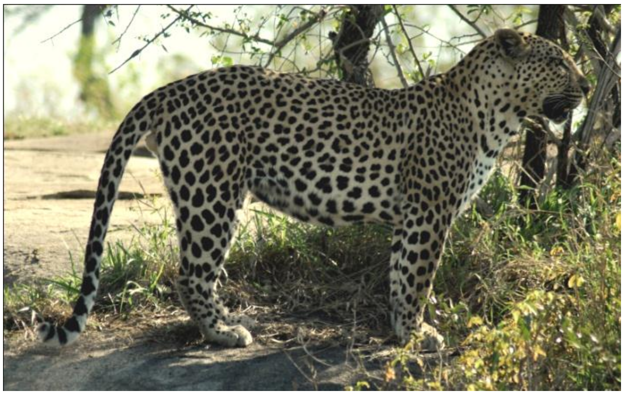

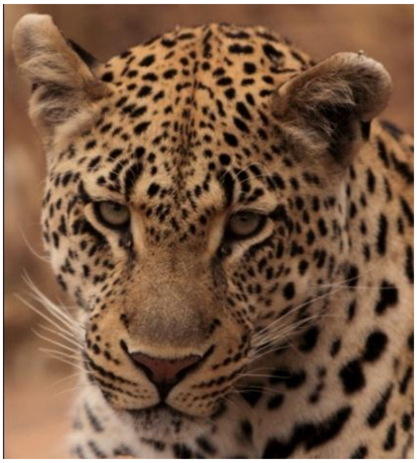

49)

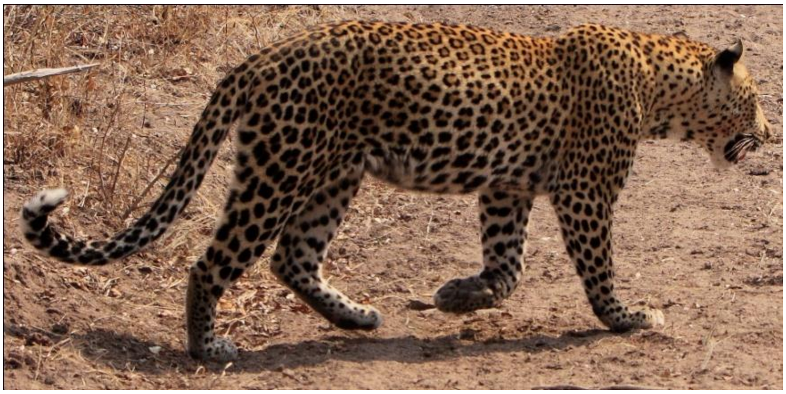

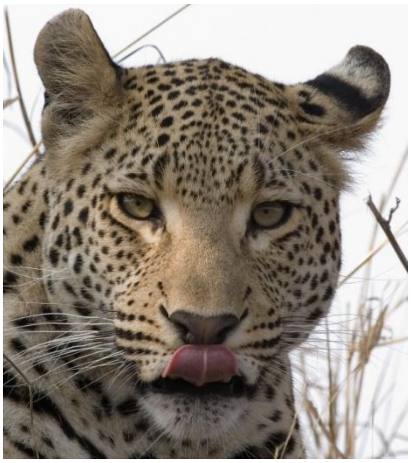

50)

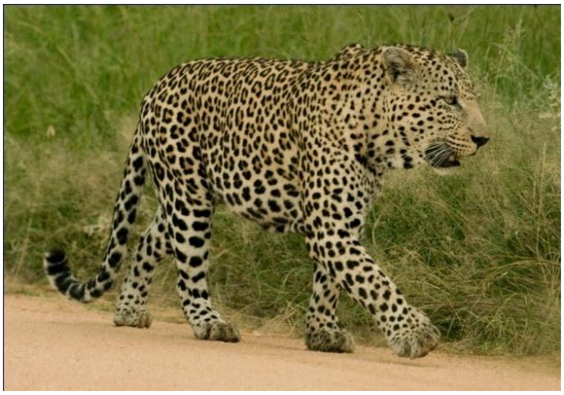

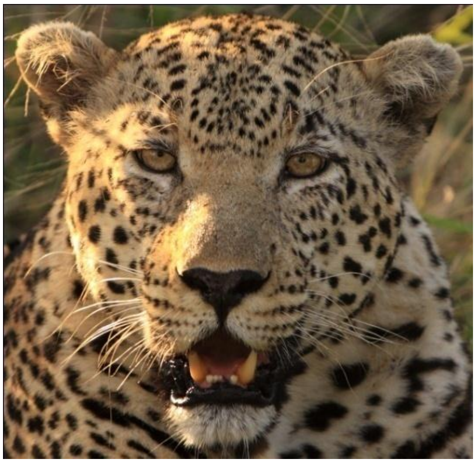

51)

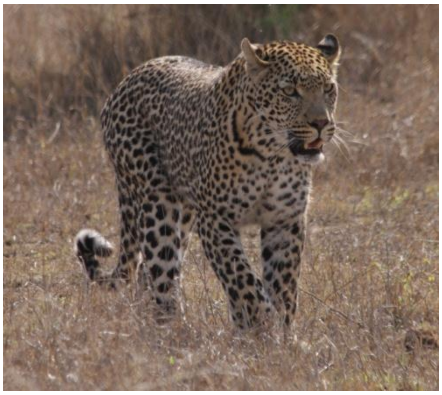

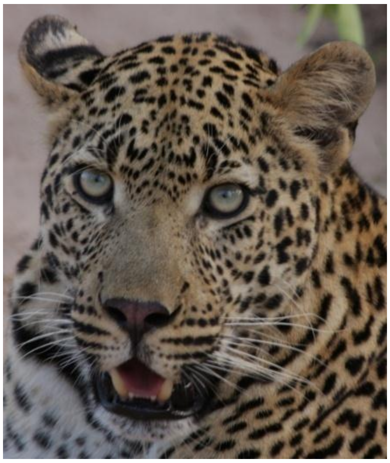

CREDITS

This test was made possible by long-term monitoring of known-aged leopards by field guides in the Sabi Sand Game Reserve, South Africa. The photographs are the property of the individual photographers and are protected by copyright.

Photographs by:

© Andrew Bachelor - 5, 10, 11, 23, 27, 29, 30, 33, 41, 49, 50
© Ben Coley - 1, 42
© Cameron Appel - 4, 9, 14, 22
© Chase Crampton - 12, 47, 51
© Christian Sperka - 8
© Darred Joubert - 2, 17
© David Dampier - 16, 18, 26, 28, 32, 35, 49
© David Pusey - 45, 46
© Duncan Gordon - 31
© Gary Parker - 19, 39, 45
© Graham Dyer - 43
© Heinrich Röntgen - 13, 46
© Jono Booth - 50
© Jono Harper - 48
© Natasha de Woronin - 3, 21, 25, 34, 36, 38, 40
© Nicky Silberbauer - 6, 7, 15, 20, 37
© Stephanie Mast - 24, 44, 48
© Talley Smith - 47
