# Supplementary figures and images for: Applicability of Age-Based Hunting Regulations for African Leopards
Source: PLoS One. 2012 Apr 6;7(4):e35209. doi: 10.1371/journal.pone.0035209 (PMC3320874; doi:10.1371/journal.pone.0035209)

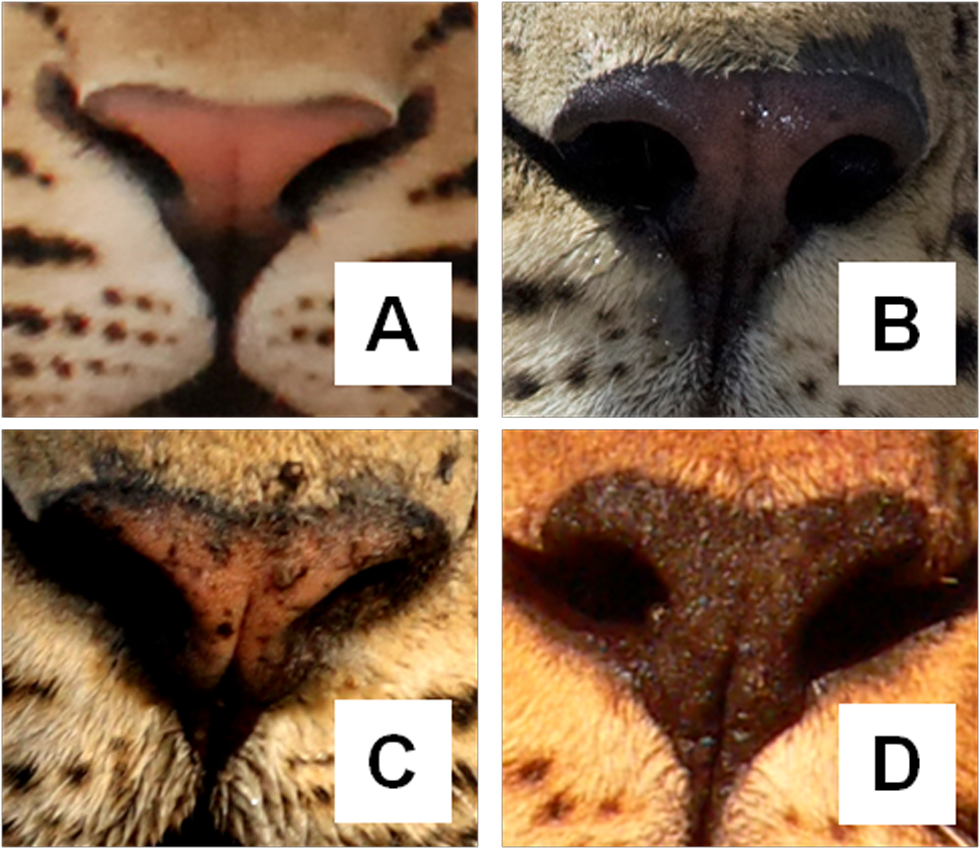

Supplement: Figure S2 — Examples of nose colour categories used in the age determination analyses. (A) 11-month male: nose colour category = pink; (B) 2.8-year male: nose colour category = pink-grey; (C) 5.3-year male: nose colour category = pink-spotted; (D) 9.0-year male: nose colour category = black. (TIF) [file pone.0035209.s002.tif]

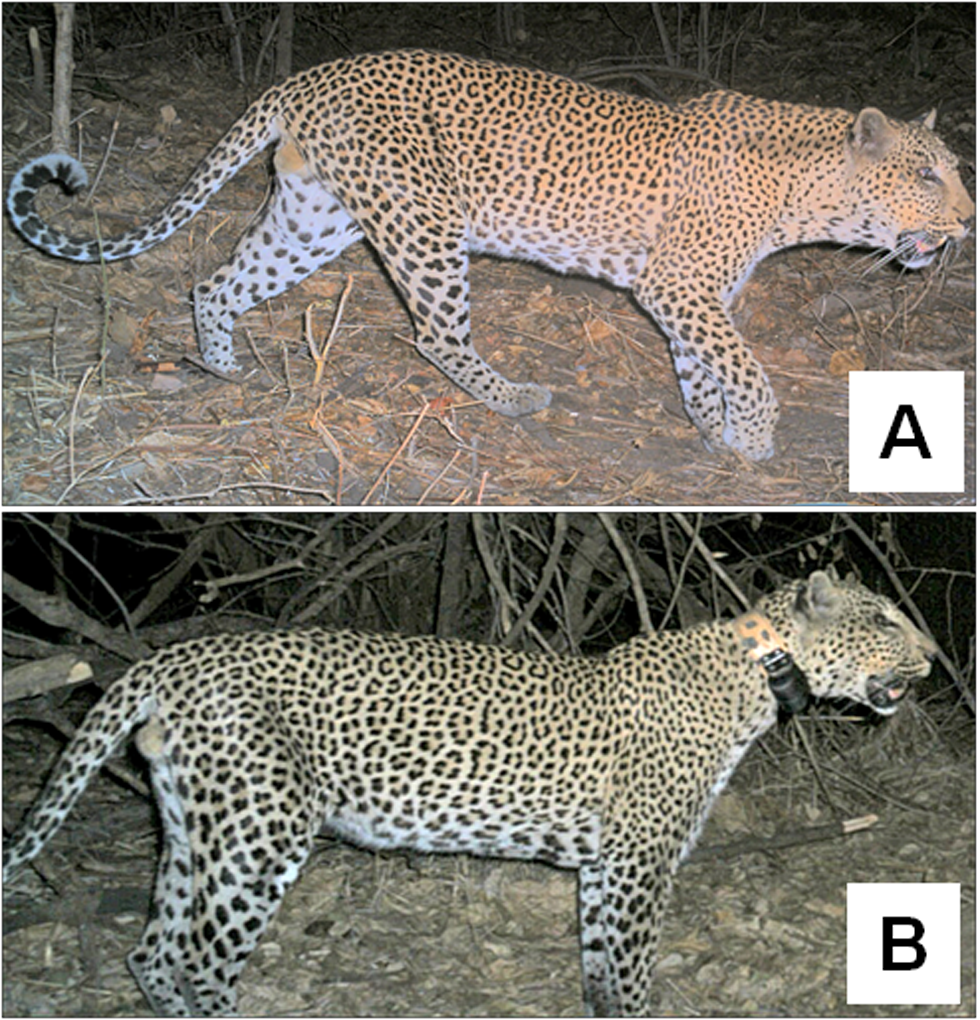

Supplement: Figure S3 — Effect of body condition on dewlap size in male leopards. The same individual male leopard camera-trapped in July 2009 (A) and August 2010 (B) in Niassa National Reserve, Mozambique (Photo credits: Niassa Carnivore Project). It is unknown what caused the deterioration in condition. (TIF) [file pone.0035209.s003.tif]

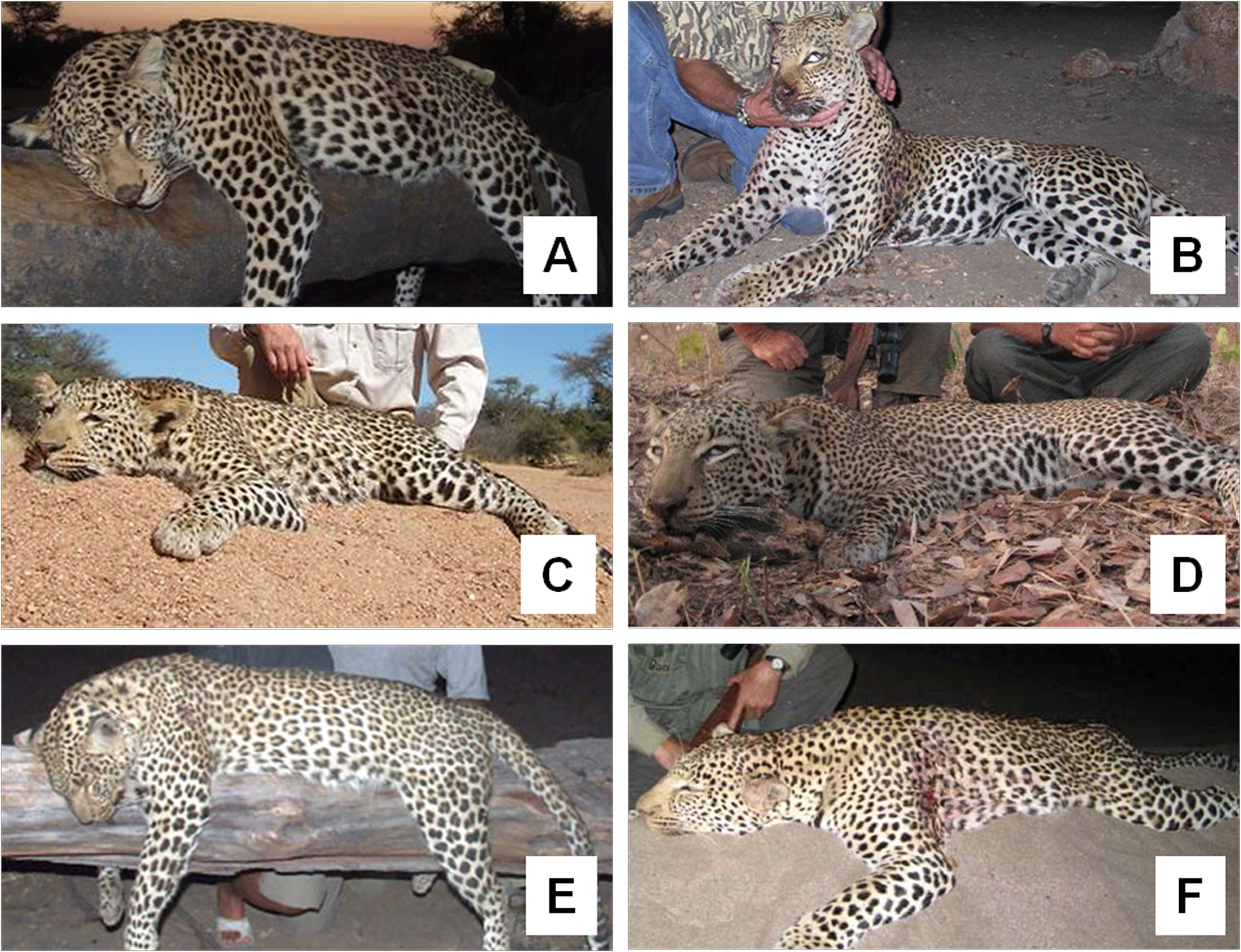

Supplement: Figure S4 — Examples of leopard trophies from different countries exhibited on hunting company websites that are likely females or <2 year males. (A) Botswana; (B) Mozambique; (C) Namibia; (D) Tanzania; (E) Zambia; (F) Zimbabwe. (TIF) [file pone.0035209.s004.tif]
