# Supplementary material for: Applicability of Age-Based Hunting Regulations for African Leopards
Source: PLoS One. 2012 Apr 6;7(4):e35209. doi: 10.1371/journal.pone.0035209 (PMC3320874; doi:10.1371/journal.pone.0035209)
Supplement: Figure S5 — Protocol for collecting data from trophy hunted lion and leopard. (TIF) [file pone.0035209.s005.tif]

PHOTOGRAPHS REQUIRED FROM THE CLEANED SKULL OF EACH TROPHY

The same photographs are required for lion and leopard.

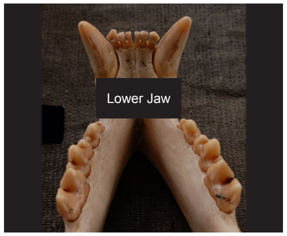

1. Lower jaw showing all the teeth and chipping of the enamel ridge on the back of the canines.

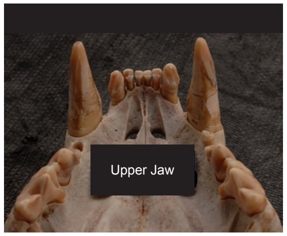

2. Upper jaw showing all the teeth and chipping of the enamel ridge on the back of the canines.

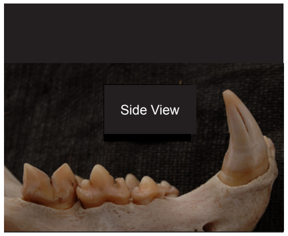

3. Side view of the lower jaw (either side) showing the canine and wear on the cusps of molars and premolars.

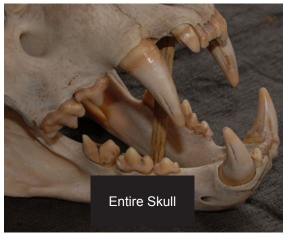

4. Wide shot of all the teeth showing wear, broken teeth, and teeth coloration.

REMOVAL OF PREMOLAR P^2

X-rays of teeth can assist with aging trophies as the pulp cavity fills in as lion and leopard get older. The best tooth to remove is the first upper premolar (P2); it does not matter from which side of the skull. P2 is small and its removal will not affect the overall appearance of the trophy. The tooth can be placed in a labeled envelope and sent with the hunt return form and photographs.

LION SKULL SHOWING THE POSITION OF PREMOLAR P^2 TO BE REMOVED FOR X-RAY ANALYSIS

The same tooth is removed for lion and leopard.

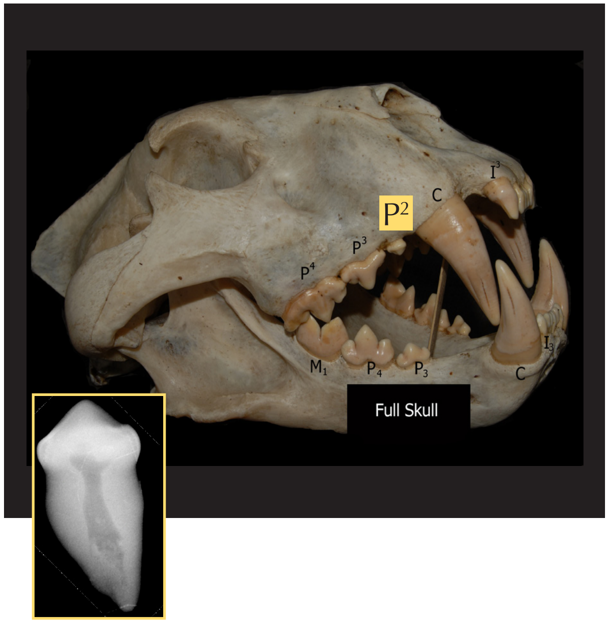

OPTIONAL DATA

COLLECTED AT HUNTER'S DISCRETION

1. Tissue samples for genetic analysis:
Genetic material can be collected from trophies by cutting a small (3-4 mm) disc of skin (that includes both tissue and hair) from anywhere on the carcass. This can be stored in a sterile container filled with 95% ethanol or salt. Alternatively, several hairs can be plucked from the trophy (with hair root attached) and placed in a labeled paper envelope.

2. Blood samples for disease screening:
Blood samples can be collected by placing a Whatman blotting paper on the bullet wound until it has soaked through. The paper is air-dried for 5-10 minutes and stored in a labeled envelope in a cool place.

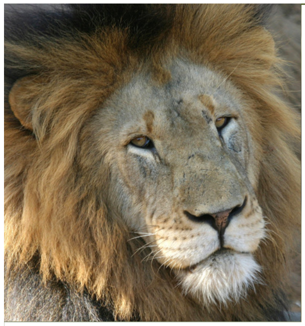

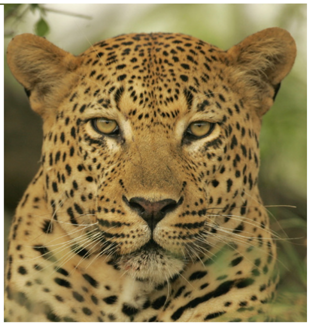

COLLECTING DATA

FROM SPORT HUNTED LION & LEOPARD TROPHIES

Wild lion and leopard populations are sensitive to human disturbance and have recently suffered widespread declines. Sport hunting can provide considerable benefits to their conservation but it must be managed sustainably in line with 'best practice' standards. Detailed information on hunting effort, trophy age, and trophy quality can help wildlife authorities track population trends of hunted species over time and allows them to react to changes accordingly. However, this is only possible if the data are collected in a rigorous & consistent manner, which requires buy-in from the hunting community. Here we provide guidelines on the information and measurements that should routinely be recorded from every trophy animal and each hunt.

PANTHERA
LEADERS IN WILD CAT CONSERVATION

NIASSA CARNIVORE PROJECT

HUNT RETURN FORMS

Authorities typically require operators to submit a hunt return form that includes information on hunt success and trophy quality. However, additional information on hunt effort is needed and it is essential that operators submit a form after every lion and leopard hunt (i.e. for successful and unsuccessful hunts).

MEASUREMENTS

The age of harvested individuals may influence the biological impacts of hunting and accurate measurements of body and skull size can help authorities to reliably age trophies.

The following measurements are required for lion and leopard trophies before they are skinned:

- Body length (cm, tip of nose to tip of tail; Measurement A–B)
- Shoulder height (cm, tip of scapula to back of plantar pad; Measurement C)
- Neck circumference - only for leopard (cm, immediately behind the ear; Measurement D)

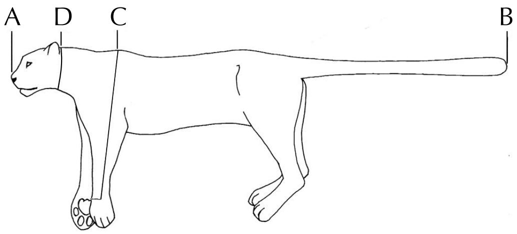

FIGURE 1: Body measurements to be taken for lion and leopard trophies before skinning.

MEASUREMENTS (CONTINUED)

The following measurements are required from the cleaned skull of each trophy:

- Skull length (mm, greatest length of skull, measured as a straight line between pegs)
- Skull width (mm, greatest width of skull, measured across zygomatic arches)

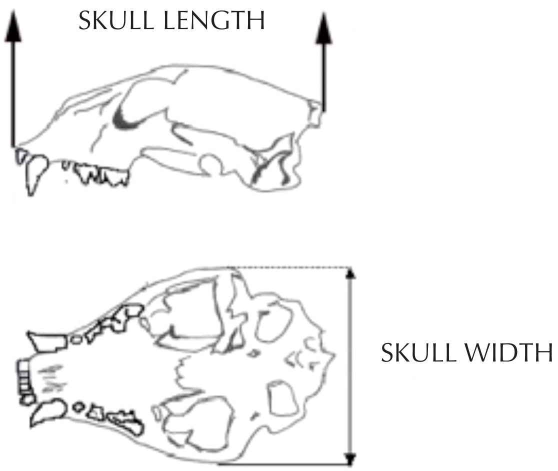

FIGURE 2: Measurements to be taken from the cleaned skull of each lion and leopard trophy.

PHOTOGRAPHS

Detailed photographs of trophies can also be used to age lion and leopard. They must be taken from the correct angle and at the highest possible resolution (minimum of 1MB). Nose, teeth, and skull photographs are best taken with the macro-function setting to prevent blurring. Digital images must be labeled with the relevant permit number (as appears on the hunt return form) and the name of the operator, client, and hunting block/property where the hunt was undertaken. The time and date stamp must be set correctly.

PHOTOGRAPHS REQUIRED FROM EACH TROPHY BEFORE IT IS SKINNED

The same photographs are required for lion and leopard except for #6, which is only necessary for leopard.

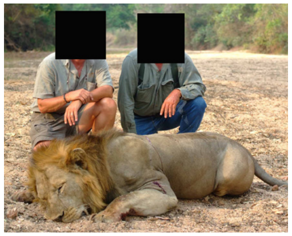

1. Side view showing the entire body with the hunter positioned directly behind for scale. Useful for assessing body size and condition, and mane development in lions.

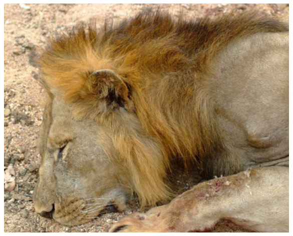

2. Side view of the head, neck and shoulders showing mane development in lions and dewlap development in leopards (the head must be lifted).

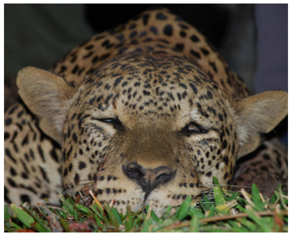

3. Frontal view of the face showing the condition and position of the ears, and facial scarring.

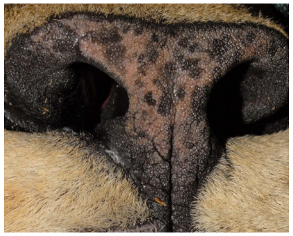

4. Close up of the nose clearly showing the pigmentation.

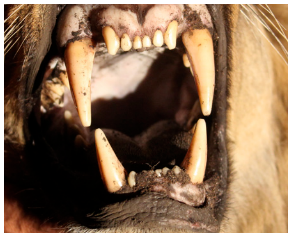

5. Frontal view of the teeth showing coloration and wear on the canines and incisors.

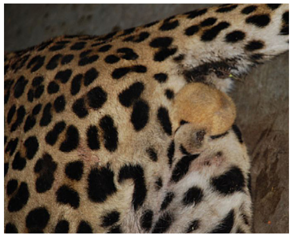

6. **Hindquarters** showing the scrotum (only required for leopard).
