# Supplementary material for: Applicability of Age-Based Hunting Regulations for African Leopards
Source: PLoS One. 2012 Apr 6;7(4):e35209. doi: 10.1371/journal.pone.0035209 (PMC3320874; doi:10.1371/journal.pone.0035209)
Supplement: Table S1 — Results of generalized linear models assessing the likelihood of professional hunters correctly identifying the sex of leopards in survey photographs. (DOC) [file pone.0035209.s006.doc]

**Table S1.** Results of generalized linear models assessing the likelihood of professional hunters correctly identifying the sex of leopards in survey photographs.

| **Predictor** | **Wald chi-squared** | **Degrees of freedom** | ***P*** |
| --- | --- | --- | --- |
| Leopards hunted | 0.547 | 2 | 0.761 |
| Leopard age class | 140.397 | 3 | <0.001 |
| Photograph presentation | 0.043 | 2 | 0.835 |
