# Supplementary material for: Applicability of Age-Based Hunting Regulations for African Leopards
Source: PLoS One. 2012 Apr 6;7(4):e35209. doi: 10.1371/journal.pone.0035209 (PMC3320874; doi:10.1371/journal.pone.0035209)
Supplement: Table S3 — Morphological measurements of radio-collared leopards from the Phinda-Mkhuze Complex (PMC) in northern KwaZulu-Natal, South Africa [19] . (DOC) [file pone.0035209.s008.doc]

**Table S3.** Morphological measurements of radio-collared leopards from the Phinda-Mkhuze Complex (PMC) in northern KwaZulu-Natal, South Africa [19].

|  | **Sex and age class** | | | | | | | | | | | | | | |
| --- | --- | --- | --- | --- | --- | --- | --- | --- | --- | --- | --- | --- | --- | --- | --- |
|  | **Female ≥4 years**  **(n = 11)** | | | **Male <2 years**  **(n = 10)** | | | **Male 2-3 years**  **(n = 15)** | | | **Male 4-6 years**  **(n = 10)** | | | **Male ≥7 years**  **(n = 4)** | | |
| **Parameter** | **Mean** | **SE** | **Range** | **Mean** | **SE** | **Range** | **Mean** | **SE** | **Range** | **Mean** | **SE** | **Range** | **Mean** | **SE** | **Range** |
| Age (months) | 68.13 | 6.71 | 48-111 | 15.35 | 1.15 | 1-20 | 29.20 | 1.86 | 24-45 | 60.30 | 2.91 | 48-78 | 111.00 | 15.20 | 84-150 |
| Weight (kg) | 37.62 | 1.06 | 34-45 | 37.80 | 1.83 | 32-38 | 51.10 | 2.24 | 26-60 | 66.10 | 2.25 | 56-79 | 72.25 | 1.25 | 69-75 |
| Body length (cm)^a^ | 182.75 | 1.22 | 175-189 | 187.10 | 2.31 | 174-197 | 196.80 | 2.92 | 183-216 | 214.20 | 1.28 | 209-220 | 218.00 | 5.02 | 203-224 |
| Tail length (cm) | 78.05 | 0.59 | 75-81 | 79.95 | 0.96 | 76-84 | 87.00 | 0.71 | 80-92 | 87.40 | 1.65 | 79-95 | 87.55 | 0.54 | 87-89 |
| Shoulder height (cm) | 57.71 | 1.04 | 53-64 | 58.05 | 1.07 | 50-61 | 62.73 | 1.07 | 52-68 | 68.43 | 0.82 | 64-62 | 71.43 | 1.48 | 69-75 |
| Chest girth (cm) | 65.01 | 0.96 | 60-71 | 65.20 | 1.10 | 60-70 | 71.37 | 1.41 | 56-78 | 79.10 | 0.82 | 76-84 | 86.63 | 2.93 | 81-94 |
| Neck circumference (cm) | 41.21 | 0.91 | 37-48 | 41.66 | 0.95 | 38-48 | 46.21 | 0.95 | 38-52 | 53.75 | 1.30 | 47-60 | 58.43 | 1.28 | 55-61 |
| Head length (cm) | 25.53 | 0.28 | 24-27 | 26.99 | 0.58 | 24-31 | 28.75 | 0.52 | 22-31 | 29.77 | 1.09 | 21-33 | 32.13 | 0.43 | 31-33 |
| Head circumference (cm) | 43.58 | 0.32 | 43-46 | 43.93 | 0.76 | 41-48 | 48.04 | 0.80 | 43-53 | 55.29 | 0.78 | 51-59 | 59.13 | 0.75 | 57-61 |

^a^ Measured as a straight line from the tip of the nose to the end of the tail

Environmental conditions are similar in the PMC and Sabi Sand GR. Leopard ages were estimated using wear and eruption of teeth [20], the aging criteria identified in this study, and associated behaviour of individuals [7]
